# Assessment of the psychometrics properties of the Senegalese kidney disease quality of life – 36 (KDQOL-36) in patients with chronic kidney disease

**DOI:** 10.1080/1179271X.2026.2688843

**Published:** 2026-06-22

**Authors:** Moustapha Faye, Abdou Omorou, Luc Frimat, Jonathan Epstein, Adama Sow, Ramatoulaye Abdoulahi Niang, Abdou Niang, Elhadji Fary Ka, Adama Faye, Francis Guillemin

**Affiliations:** a Service de Néphrologie – CHU Aristide Le Dantec, Université Cheikh Anta Diop, Dakar-Fann, Sénégal; b CHRU-Nancy, Inserm, Université de Lorraine, CIC, Épidémiologie Clinique, Nancy, France; c Université de Lorraine, Inserm, INSPIIRE, Nancy, France; d Service de Néphrologie – CHU Dalal Jamm, Université Cheikh Anta Diop, Dakar-Fann, Sénégal; e Institut Santé et développement (ISED), Université Cheikh Anta Diop, Dakar-Fann, Sénégal

**Keywords:** Validity, reliability, KDQOL-36, health-related quality of life, chronic kidney disease, Senegal

## Abstract

**Objectives:**

This study aimed to assess the validity, reliability and responsiveness of the Senegalese kidney disease quality of life (KDQOL-36).

**Methods:**

This cross-sectional and multicenter psychometric evaluation was conducted between June and November 2024. The Wolof version of the KDQOL-36 scores was assessed in 575 Senegalese patients aged 18 years or older with stage 3–4 CKD or on dialysis, who had been followed for at least three months and not hospitalized during the previous three months. The factor structure was evaluated using confirmatory factor analysis. We assessed internal consistency and test–retest reliability using α and ω coefficients, the intraclass correlation coefficient (ICC) and Bland–Altman analysis. Construct validity was examined using “known group analyses”, and responsiveness was evaluated using the paired Wilcoxon test, standardized response means and effect size.

**Results:**

A bifactor structure was retained for the generic module, comprising overall, physical and mental health. A three-factor structure was confirmed for the kidney disease targeted scale, including the burden, symptoms/problems and effects. The scale and subscales showed sufficient internal consistency. Test–retest reliability was moderate to good. Known group analyses revealed significant differences in the scale scores between patients who received dialysis and who did not receive dialysis, and between men and women. Overall health, physical health, symptoms/problems and effects demonstrated moderate to high sensitivity to change.

**Conclusions:**

This study provides evidence supporting the structural validity, construct validity, reliability, and responsiveness of the Senegalese Wolof version of the KDQOL-36 for assessing health-related quality of life.

## PLAIN LANGUAGE SUMMARY

Chronic kidney disease (CKD) can affect many aspects of daily life, including physical health, emotional well-being, symptoms, and social activities. Measuring these effects helps healthcare professionals better understand patients’ experiences and evaluate treatments. The Kidney Disease Quality of Life-36 (KDQOL-36) questionnaire is one of the most widely used tools for this purpose, but it had not previously been validated for use in Senegal. We translated and culturally adapted the KDQOL-36 into Wolof, the most widely spoken language in Senegal, and tested it in 575 adults with CKD, including patients receiving dialysis and those not yet on dialysis. We examined whether the questionnaire accurately measured quality of life, produced consistent results, and was able to detect changes in patients’ health over time. The results showed that the Senegalese Wolof version of the KDQOL-36 is a reliable and valid tool for assessing quality of life in people with CKD. The questionnaire successfully distinguished between groups known to have different levels of health-related quality of life, such as dialysis and non-dialysis patients, and men and women. Most of its scales showed good reliability, and several were sensitive to clinical improvement over time. These findings support the use of the Senegalese Wolof KDQOL-36 in clinical research and may help healthcare professionals better evaluate the impact of CKD and its treatments on patients’ lives.

## Introduction

In Senegal, as in many parts of the world, chronic kidney disease (CKD) represents a significant public health issue because of its prevalence (4.3%–4.9%) [1,2], elevated mortality [3], considerable cost [4,5], and complex management. [6] CKD has a substantial negative impact on health-related quality of life (HRQOL) [7–12], due to the disease itself and its comorbidities, as well as the burden and symptoms associated with its treatments, including conservative care and dialysis. However, the past decade has seen notable progress in CKD care in Senegal, with increased availability and accessibility of both conservative treatment and kidney replacement therapies [13,14]. As a result, patients are living longer, drawing increased attention to perceived health outcomes, particularly HRQOL.

Quality of life is defined by the World Health Organization (WHO) as “individuals’ perceptions of their position in life in the context of the culture and value systems in which they live and in relation to their goals, expectations, standards and concerns” [15]. In addition to traditional outcomes such as morbidity and mortality, HRQOL is increasingly recognized as a key clinical endpoint by patients, health care professionals, and funding stakeholders [16,17].

Given its importance as a clinical endpoint in nephrology, the measurement of HRQOL has become essential. The instruments used to measure it, commonly known as patient-reported outcome measures (PROM), must be adapted to the cultural context in which they are applied, and meet rigorous standards of validity, reliability, and reproducibility [16,18]. Despite this need, very few PROMs have been adapted and validated for CKD patients in Africa [19].

Several HRQOL instruments have been developed, including generic tools, which allow for comparisons across different populations and conditions [15,20–22], and disease-specific tools tailored to particular health conditions such as CKD [21,23].

Among these, the KDQOL-36 is widely used and incorporates both a generic core, namely, the Short Form-12 (SF-12) developed by Ware et al. [24] that includes 12 items, and a kidney disease targeted subscale (KDS) with 24 items [25]. To ensure their relevance and interpretability, such instruments must undergo a process of cross-cultural adaptation followed by psychometric validation in the intended setting [26].

The cross-cultural adaptation of the KDQOL-36 into the Wolof language, tailored to the sociolinguistic and cultural specificities of the Senegalese population, was previously conducted (article submitted) in accordance with Epstein’s recommendations [27]. In Senegal, Wolof is the main vehicular language. It is spoken by more than 70% [28] of the Senegalese population and serves as an interethnic communication language among speakers of different native languages [29]. Building on that initial work and in view of the clinical importance of HRQOL in CKD, we undertook the psychometric validation of the Senegalese Wolof version of the KDQOL-36. The aim of the present study was to assess its validity, reliability and responsiveness using classical test theory (CTT) methods.

## Methods

### Study design and sample

A cross-sectional study was performed from June to November 2024 in nephrology and dialysis units across the Dakar and Thiès regions of Senegal. Participants were recruited using a nonprobability convenience sampling approach. Eligibility criteria included being over 18 years of age; living with stage 3, 4, or 5 CKD on dialysis according to the Kidney Disease Improving Global Outcomes (KDIGO) classification [30]; being followed for CKD for more than three months; and not having been hospitalized in the past three months. Among a source population of 662 patients who visited these units during the study period, 575 met the inclusion criteria and were included (Figure S1).

### KDQOL-36 questionnaire and scoring

The KDQOL-36 is a short form that includes the SF-12 as a generic core plus the burden of kidney disease (BKD), symptoms/problems of kidney disease (SKD), and effects of kidney disease (EKD) scales from the KDQOL-SF^™^v1.3. [31] The translation of the KDQOL-36 into the Wolof language and the cross-cultural adaptation to the Senegalese context were performed in accordance with Epstein’s recommendations (Supplementary material, Text S1) [27].

The generic module, which includes the first 12 items of the questionnaire, corresponds to the SF-12. It is a self-administered questionnaire composed of 12 statements with Likert-type response options ranging from 3 to 5 levels of agreement, frequency, or intensity [24]. It measures two summary scores: The physical component summary (PCS) and the mental component summary (MCS). Several factorial structures have been described in the literature, including the classical correlated two-factor structure [21,23] and a bifactor model including one general factor and two specific factors [32,33].

The KDS, which comprises the last 24 items of the questionnaire, is also a self-administered questionnaire with Likert-type responses in the form of questions, offering 5 levels of frequency, or intensity. This module is structured into three correlated dimensions: BKD (Items i13 – i16), SKD (Items i17 – i28), and EKD (Items i29 – i36). A bifactorial structure was reported by Peipert et al. in the US population [34].

The KDQOL^™^-36 scoring program developed by RAND Health Care was used for scoring PCS, MCS, BKD, SKD, and EKD [31].

### Data collection

Consenting patients were invited either to self-complete the paper-based Senegalese version of the KDQOL-36 or to complete it through an interviewer-administered format conducted by trained research assistants. A retest was administered 15 to 20 days after the initial interview on a convenient subsample of 167 patients. At the retest, patients were asked whether their clinical condition had deteriorated, remained the same, or improved. If they had improved or deteriorated, the degree of change was quantified using a seven-point Likert scale with the following responses: “A little better,” “Somewhat better (or worse),” “Moderately better (or worse),” “Quite a bit better (or worse),” and “Very much better (or worse)” [35]. Clinical data, such as age, sex, CKD stage, duration on dialysis, dialysis center and modality, vascular access type, education level, marital status, occupation, and diabetes mellitus status, were extracted from medical records.

### Statistical analysis

The characteristics of the patients are described as numbers (percentages) or means (±SDs) and/or medians (interquartile ranges [IQRs]). The included and nonincluded patients were compared by standard statistical tests.

The structural validity of the KDQOL-36 was assessed by confirmatory factor analysis (CFA) with weighted least squares mean and variance (WLSMV) adjusted estimation.

For the generic subscale, the correlated two-factor CFA models with and without correlated errors were estimated. A confirmatory bifactor model with and without correlated errors was also constructed with a general factor (all 12 items) and 2 specific factors. For the KDS, a correlated three-factor model was estimated, with and without correlated error terms. In addition, a confirmatory bifactor model was estimated, including one general factor and three specific factors, with and without correlated errors. A comparative fit index (CFI) or Tucker-Lewis index (TLI) > 0.95 OR a root mean square error of approximation (RMSEA) < 0.06 OR a standardized root mean square residual (SRMR) < 0.08 suggest sufficient structural validity [26]. The CFA was performed using the *lavaan* package in R [36]. In addition, the bifactor model was evaluated using several other statistical indices, including hierarchical omega (ω_H_), subscale omega (ω_S_), construct reliability (H), factor determinacy (FD), explained common variance (ECV), and the average relative parameter bias (ARBP). The definitions [37] and acceptable thresholds of the bifactor statistical indices are shown in Table S1.

The internal consistency reliability for each scale and subscale was considered acceptable if the Cronbach’s alpha (*α*) and total omega (*ω_T_*) coefficient values greater than 0.70 [26]. Test–retest reliability was evaluated using the intraclass correlation coefficient (ICC) based on a two-way random-effect model with a consistency definition and single measurement unit [38] and Bland–Altman plots [39] among 74 patients who rated their clinical status as stable.

The ICCs were interpreted as follows: excellent (> 0.90), good (0.75–0.90), moderate (0.50–0.75), and poor (< 0.50) [40].

Ceiling (percentage of respondents with scores of 100) and floor (percentage of respondents with score of 0) effects should be less than 20% to ensure that the scale captures the full range of potential responses within the population.

A known-group analysis was conducted to assess the ability of the questionnaire to discriminate between patients with stage 3–4 CKD and those on chronic dialysis, as well as between male and female patients. Stage 3–4 CKD patients tend to report better HRQOL compared with those undergoing chronic dialysis, and that men generally report better HRQOL compared with women [7,9, 11,12]. Group differences were evaluated using the Wilcoxon rank-sum test.

Responsiveness was assessed among 47 patients whose clinical condition improved moderately to very much. Responsiveness is considered to be statistically significant when the p- value is < 0.05 (paired Wilcoxon test). Its magnitude is considered moderate when the standardized response mean (SRM) or effect size (ES) is > 0.5 and high when it is > 0.8 [41].

All the statistical analyses were performed in R version 4.5.0 with several packages [36,38,42,43]. A *p*-value less than 0.05 was considered to indicate a significant difference.

### Ethical considerations

This study was approved by the *Comité National d’Ethique de la Recherche en Santé* (CNERS) under registration number 00000203, dated August 12, 2024. Administrative authorization was obtained from all patient inclusion sites. All patients provided informed consent to participate in the study after receiving an information letter.

## Results

### Sample characteristics

This study included a total of 575 patients, of whom 78% were on chronic dialysis, with a median dialysis duration of 53 (IQR: 36–84) months. The median patient age was 55 (44–66) years, and 53.9% were male. The retest was conducted in 167 patients. Only 0.3% of participants completed the questionnaire by self-administration. The patients’ clinical characteristics are presented in Table 1 **(**Clinical and sociodemographic characteristics of the 575 CKD patients included in the study). Comparisons of the included and nonincluded patients are shown in Table S2.

**Table 1.** Clinical and sociodemographic characteristics of the 575 CKD patients included in the study.

Characteristics	% of missing data	Median (IQR) or number (%)
Patient’s age (in years)	0.0	55 (44–66)
Diabetes mellitus	2.3	108 (19.2)
Auto administration	0.3	6 (1.0)
Patient’s sex	0.3	
Female		266 (46.4)
Male		309 (53.9)
Education level (in years)	1.2	
0, No formal instruction		148 (26.0)
[1–6], Primary school		113 (19.9)
[7–10], Junior secondary school		83 (14.6)
[11–13], Senior secondary school		108 (19.0)
> 13, University		116 (20.4)
Patient’s occupation	9.6	
Formal occupation		111 (21.3)
No formal occupation		129 (24.8)
Without occupation		280 (53.4)
Marital status	3.0	
Single		72 (12.9)
Divorced		39 (7.0)
Married		399 (71.5)
Widowed		48 (8.6)
CKD G stages	0.0	
CKD stage 3–4		127 (22.1)
Stage 5 on dialysis		448 (77.9)
Dialysis technic	0.0	
Peritoneal dialysis		11 (2.5)
Hemodialysis		437 (97.5)
Dialysis duration (in months)	4.0	53 (36–84)
Dialysis center	0.2	
Clinique ABC		117 (26.2)
Clinique CHD		33 (7.4)
CHN de PIKINE		47 (10.5)
CHR de THIES		50 (11.2)
CHU Aristide le Dantec		89 (19.9)
CHU Dalal Jamm		19 (4.3)
Hôpital militaire de Ouakam		53 (11.9)
Hôpital Roi Baudoin		39 (8.7)
Dialysis session number per week	4.6	
< 3		46 (10.8)
≥ 3		381 (89.2)
Hemodialysis vascular access	5.1	
Temporary catheter		25 (5.9)
Permanent catheter		99 (23.3)
Native arteriovenous fistula		294 (69.2)
Graft arteriovenous fistula		7 (1.6)
Retest		167 (29.0)
Clinical conditions at retest		
Unmodified		74 (44.3)
Improved		83 (49.7)
Worsened		10 (6.0)

**Abbreviations**: IQR, interquartile range; ABC, Alioune Badara Cisse; CHD, centre d’hémodialyse de Dakar; CHN, centre hospitalier national; CHR, centre hospitalier régional; CHU, centre hospitalier universitaire.

### Items description and data quality

All response categories were used for all the items. Only Items i28 (Problems with your access/catheter site) and i35 (Your sex life) had more than 5% missing data. The items response descriptions, including missing data, are shown in Table 2 **(**Clinical and sociodemographic characteristics of the 575 CKD patients included in the study). No score showed a ceiling effect, whereas only the score “burden of kidney disease” showed a floor effect (23.24%).

**Table 2.** Clinical and sociodemographic characteristics of the 575 CKD patients included in the study.

Items	Response categories (%)					Missing (%)
	1	2	3	4	5	
General health (i1)	4.3	4.0	21.2	51.0	19.1	0.3
Moderate activities (i2)	39.3	36.5	23.5			0.7
Climb several flights of stairs (i3)	41.7	33.4	21.7			3.1
Accomplished less, physical (i4)	23.8	18.1	25.2	14.4	18.1	0.3
Limited in kind of work, physical (i5)	25.6	20.2	21.0	13.2	19.3	0.7
Accomplished less, emotional (i6)	20.7	14.8	26.8	16.5	20.2	1.0
Did work less carefully, emotional (i7)	23.7	13.9	25.0	15.8	20.0	1.6
Bodily pain (i8)	27.5	18.3	17.4	16.9	18.8	1.2
Calm and peaceful (i9)	17.9	24.3	38.4	13.2	5.0	1.0
Energy (i10)	13.7	17.7	34.4	24.0	9.0	1.0
Downhearted and blue (i11)	7.7	9.0	19.8	21.7	40.5	1.2
Social functioning (i12)	23.5	11.7	16.5	13.6	33.9	0.9
My kidney disease interferes too much with my life? (i13)	55.3	22.6	7.1	5.4	8.3	1.2
Too much time is spent dealing with kidney disease? (i14)	55.3	25.4	5.6	4.2	8.2	1.4
I feel frustrated dealing with my kidney disease? (i15)	29.7	21.6	6.1	5.0	35.8	1.7
I feel like a burden on my family? (i16)	48.3	10.6	5.0	4.5	29.9	1.6
Soreness in your muscles? (i17)	26.3	23.8	12.9	19.5	16.3	1.2
Chest pain? (i18)	57.4	17.6	9.9	8.2	4.2	2.8
Cramps? (i19)	36.9	23.8	13.4	13.6	11.0	1.4
Itchy skin? (i20)	63.0	16.9	6.1	5.0	6.8	2.3
Dry skin? (i21)	69.6	13.7	6.6	5.6	3.0	1.6
Shortness of breath? (i22)	50.8	21.9	11.7	9.4	4.3	1.9
Faintness or dizziness? (i23)	54.3	22.6	10.1	5.2	5.9	1.9
Lack of appetite? (i24)	44.5	18.3	12.7	13.4	9.9	1.2
Washed out or drained? (i25)	36.2	25.0	14.4	14.3	7.7	2.4
Numbness in hands or feet? (i26)	56.9	17.7	8.7	11.8	3.0	1.9
Nausea or upset stomach? (i27)	66.6	16.0	8.0	5.7	2.4	1.2
Problems with your access/catheter site? (i28)	39.7	19.1	6.8	9.6	1.4	23.5
Fluid restriction? (i29)	36.3	15.0	10.8	23.8	12.9	1.2
Dietary restriction? (i30)	48.3	13.2	9.0	17.0	11.0	1.4
Your ability to work around the house? (i31)	40.2	16.0	8.9	16.9	16.0	2.1
Your ability to travel? (i32)	29.4	11.7	9.6	24.2	23.0	2.3
Being dependent on doctors and other medical staff? (i33)	62.1	8.2	7.1	15.3	5.4	1.9
Stress or worries caused by kidney disease? (i34)	47.5	18.1	10.6	13.9	8.0	1.9
Your sex life? (i35)	55.5	8.3	8.5	12.3	8.7	6.6
Your personal appearance? (i36)	45.6	18.3	13.7	15.0	6.3	1.2

### Psychometric properties

#### Structural validity

The CFA results for the generic subscale and the KDS are shown in Table S3.

**Figure 1.**
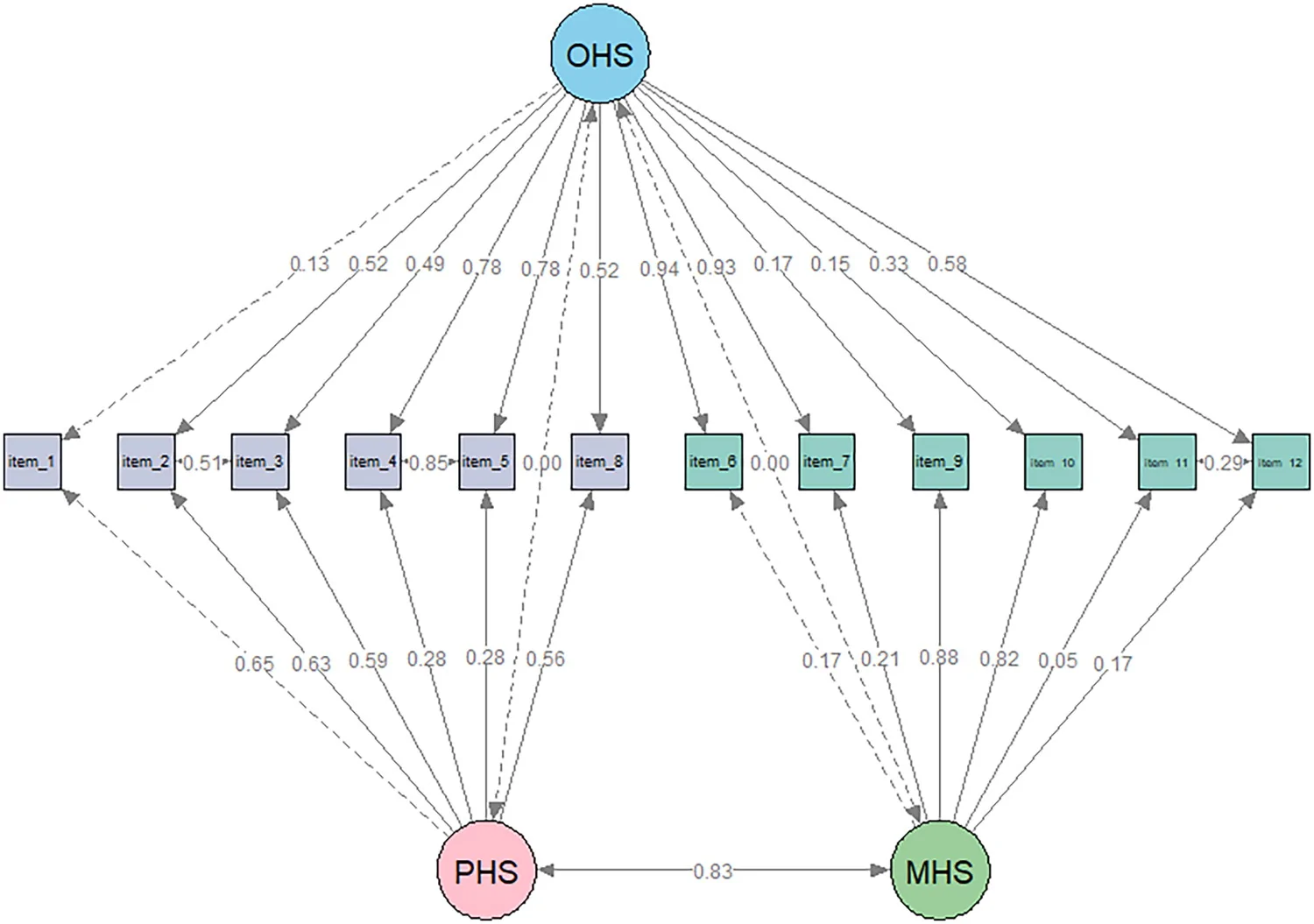
A bifactor model with correlated errors for the generic subscale of the Senegalese KDQOL-36. The general factor (OHS) is specified as orthogonal to the two specific factors: physical (PHS) and mental (MHS) health. OHS, Overall Health Scale; PHS, Physical Health Scale; MHS, Mental Health Scale.

For the generic subscale, Models 1, 2 and 3 demonstrated insufficient goodness-of-fit indices. The bifactor model with correlated error terms (Model 5) showed sufficient goodness-of-fit, with χ^2^/df = 2.9, RMSEA = 0.060 (95% CI 0.048–0.073), robust CFI = 0.975, robust TLI = 0.956 and SRMR = 0.027. All the items load strongly on either the general factor or one of the specific factors (loadings ranging from 0.56 to 0.94) (Figure 1**:** A bifactor model with correlated errors for the generic subscale of the Senegalese KDQOL-36).

This bifactor model was therefore selected. It includes a general factor comprising all 12 items, referred to as the Overall Health Scale (OHS) (ECV = 57%, ω_H_ = 0.71, H = 0.95, FD = 0.98); a specific physical factor (Physical Health Scale, PHS), including Items i1–i5 and i8 (ω_S_ = 0.33); and a specific mental factor (Mental Health Scale, MHS) including Items i6, i7, and i9–i10 (ω_S_ = 0.28). The statistical indices of the SF-12 bifactor model are presented in Table S4.

**Figure 2.**
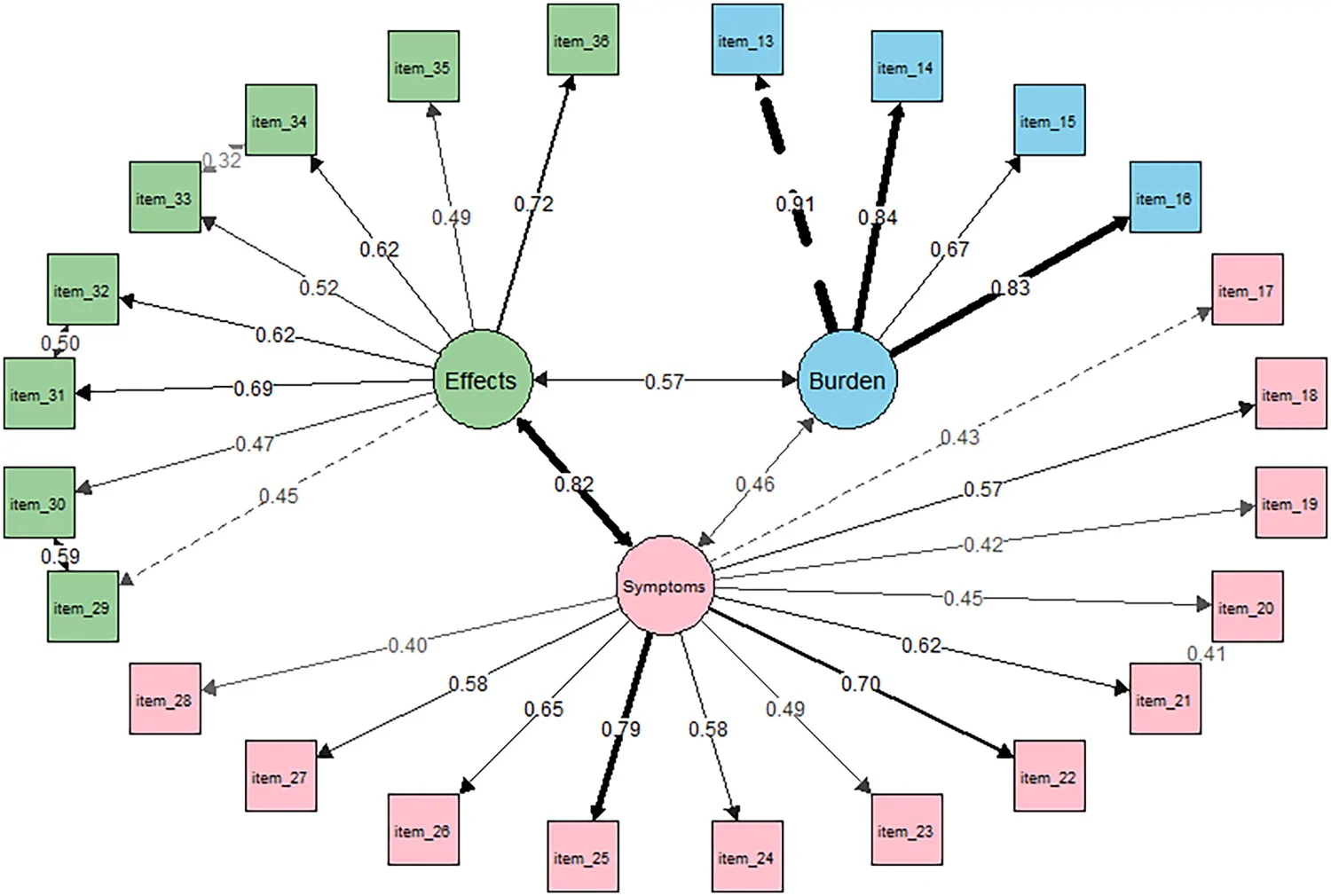
A three-factor model with correlated error terms for the kidney disease-targeted subscale of the Senegalese KDQOL-36.

For the KDS, the three-factor model (Model 1) demonstrated insufficient goodness-of-fit indices. According to the modification indices of Model 1, the respecified model (Model 2) showed sufficient goodness-of-fit, with χ^2^/df = 3.7, RMSEA = 0.069 (95% CI 0.064–0.074), robust CFI = 0.794, robust TLI = 0.768 and SRMR = 0.078. The bifactor model including a general factor and three specific factors (BKD, SKD, and EKD) showed better goodness-of-fit indices. However, the general factor score was not reliable (Table S5). The correlated three-factor model, with correlations between error terms (Model 2), was therefore selected (Figure 2**:** A three-factor model with correlated error terms for the kidney disease-targeted subscale of the Senegalese KDQOL-36).

#### Internal consistency and test–retest reliability

All the scales and subscales were sufficiently internally consistent, with Cronbach’s α and ω_T_ values exceeding 0.70 (Table 3**:** Internal consistency, floor/ceiling effects, test–retest reliability and responsiveness index of the scales and subscales of the Senegalese KDQOL-36). According to the ICCs, the BKD, SKD, EKD and PHS had good test–retest reliability, whereas the other subscales (OHS, MHS, PCS and MCS) had moderate test–retest reliability (Table 3). In the Bland–Altman analysis (Figure S2), the proportions of observations falling outside the limits of agreement were 8.5% for PCS, 7.0% for MCS, 2.9% for OHS, 8.5% for PHS, 5.6% for MHS, 9.9% for Burden, 7.9% for Symptoms and 7.2% for Effects. The scoring programs of the OHS, PHS and MHS are shown in Supplementary material, Text S2.

**Table 3.** Internal consistency, floor/ceiling effects, test–retest reliability and responsiveness index of the scales and subscales of the Senegalese KDQOL-36.

Subscales-N. items	Mean ± SD/median (IQR)	Alpha (95% CI)	ω_T_	Floor/ceiling effects, %	ICC (95% CI)	SRM/ES
Generic subscale						
OHS-12	50.7 ± 25.0	0.88 (0.86–0.89)	0.90	0.18/0.18	0.61 (0.44–0.74)	0.804/0.545
PHS-6	43.8 ± 17.9	0.82 (0.80–0.84)	0.86	0.17/0.17	0.82 (0.72–0.88)	0.779/0.622
MHS-6	52.3 ± 22.4	0.75 (0.72–0.78)	0.77	0.18/0.53	0.67 (0.51–0.78)	0.451/0.322
PCS-12	36.7 ± 11.6			0/0	0.66 (0.50–0.77)	−0.019/−0.017
MCS-12	45.0 ± 7.6			0/0	0.61 (0.44–0.74	−0.286/−0.198
KDS						
BKD-4	25.0 (6.3–50.0)	0.78 (0.75–0.81)	0.81	23.24/3.52	0.90 (0.87–0.93)	0.04/0.012
SKD-12	79.2 (64.6–88.6)	0.80 (0.77–0.82)	0.79	0.18/2.82	0.85 (0.80–0.89)	0.91/0.673
EKD-8	68.8 (50.0–84.4)	0.77 (0.74–0.79)	0.78	0.53/5.99	0.86 (0.81–0.85)	0.64/0.414

Abbreviations: IQR, Interquartile Range; SD, Standard Deviation; CI, Confidence Interval; ω_T_, omega total coefficient; ICC, Intraclass Correlation Coefficient; SRM, Standardized Response Mean; ES, Effect Size; OHS, Overall health Scale; PHS, Physical Health Scale; MHS, Mental Health Scale; PCS, Physical Component Summary; MCS, Mental Component Summary; KDS, Kidney disease targeted subscale; BKD, Burden of Kidney Disease; SKD, Symptoms/problems of Kidney Disease; EKD, Effect of Kidney Disease.

The subscales scores were standardized between 0 to 100 with the higher score indicate better quality of life.

The OHS, PHS, MHS, PCS, and MCS scores were missing in 5.4%, 4.2%, 2.4%, 2.1%, and 2.1% of cases, respectively. The scores of the KDS subscales were missing in 1.2% of cases.

#### Known group analysis

**Figure 3.**
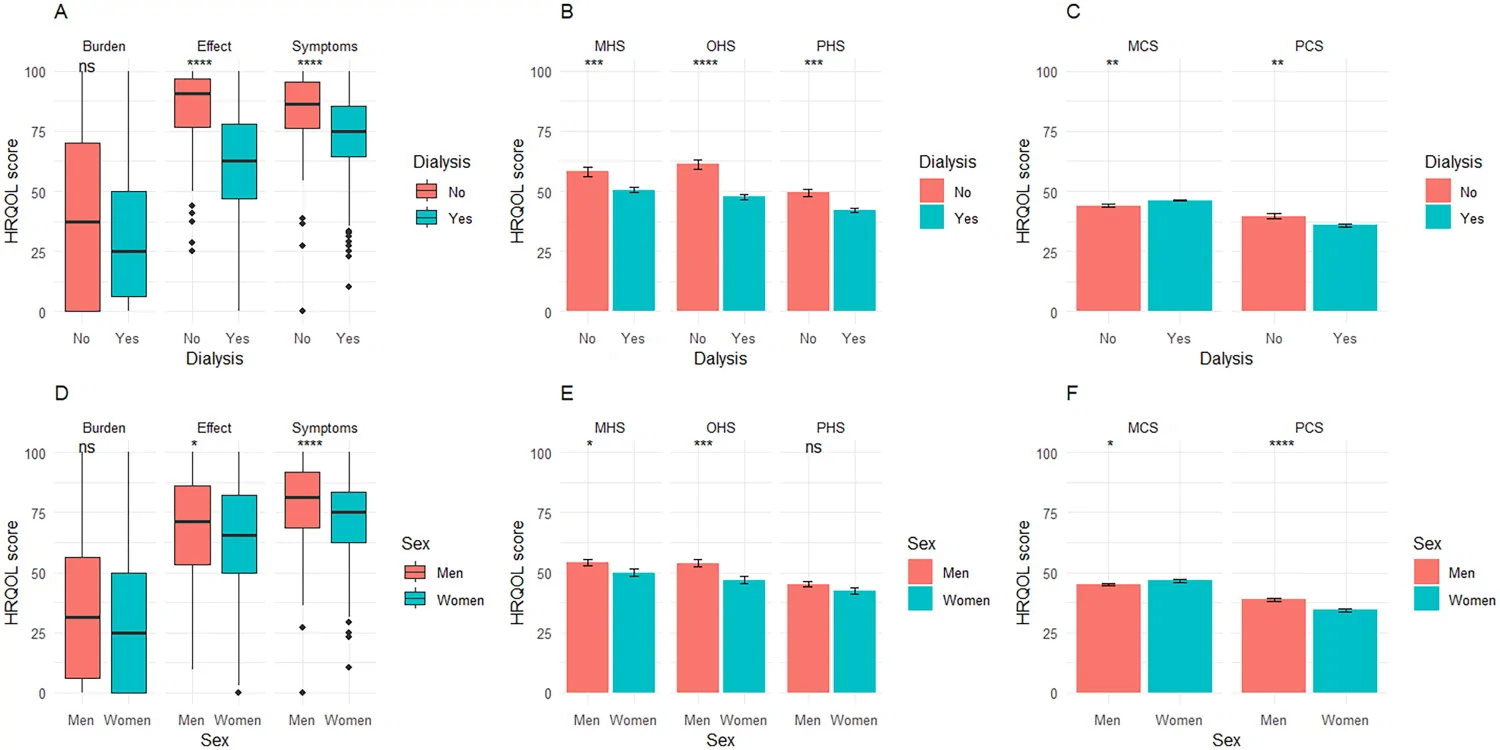
Comparison of health-related quality of life (HRQOL) scores by sex and dialysis status (known group analysis in 575 patients). HRQOL scores are expressed as standardized scores ranging from 0 to 100, with higher scores indicating better HRQOL. The *p*-value was estimated by the Wilcoxon rank sum test: ns = not significant * = *p* < 0.05; ** = *p* < 0.01; *** = *p* < 0.001; **** = *p* < 0.0001. The HRQOL scores of the generic subscale are summarized as the means + 95% confidence intervals (bar plot), whereas the burden, symptoms and effects are summarized as medians and quartiles (box plot). OHS, Overall Health Scale; PHS, Physical Health Scale; MHS, Mental Health Scale; PCS, physical component summary; MCS, mental component summary.

Our hypothesis was confirmed by known group analysis. Compared with patients with CKD stages 3–4, patients with CKD on dialysis had significantly lower scores for all the KDQOL-36 subscales except mental health (MCS) and burden. Compared with men, women had significantly lower scores for all the KDQOL-36 subscales except mental health (MCS) and burden (Figure 3**:** Comparison of health-related quality of life (HRQOL) scores by sex and dialysis status).

#### Responsiveness

According to the paired Wilcoxon test, SRM and ES, the OHS (*p* < 0.001), PHS (*p* < 0.001), SKD (*p* < 0.001), and EKD (*p* < 0.001) subscales demonstrated moderate to high sensitivity to change, whereas the PCS (*p* = 0.75), MHS (p < 0.001), MCS (*p* = 0.10), and BKD (*p* = 0.87) subscales did not (Table 3). Box plots in Figure S3 showing an increase in OHS, PHS, and symptom subscale scores at retest among patients who reported moderate to very much improvement in their clinical condition.

## Discussion

Following the content validation, as demonstrated by the translation and cultural adaptation process, the Senegalese wolof version of the KDQOL-36 showed sufficient psychometric properties.

The confirmatory two-factor model of the generic subscale demonstrated insufficient goodness-of-fit indices despite being extensively supported by prior literature in Africa [19] and others parts of the world [21,23]. The confirmatory bifactor model with a general score referred to as OHS and the two specific scales (PHS and MHS) were well adapted to our data, which is consistent with the findings of Neri et al. [33]. The bifactor model and its advantages have been discussed by several authors [44,45]. A chief goal of applying a confirmatory bifactor model is to estimate a model such that the parameter estimates on the general factor accurately reflect the relations between items and the general construct of interest while controlling for the biasing effects of multidimensionality caused by content diversity [46]. To successfully achieve this goal, the general factor must validly reflect the common variance running among all the items in a measure [46]. Items i4 (accomplished less, physical), i5 (limited in kind of work, physical), i6 (accomplished less, emotional), i7 (did work less carefully, emotional), and i12 (social functioning) are predominantly markers of overall health, with items loading ≥ 0.58 (Figure 1). Items i1 (general health), i2 (moderate activities), i3 (climb several flights of stairs) are predominantly loading in PHS, whereas Items i9 (calm and peaceful) and i10 (Energy) predominantly loading in MHS, with items loading ≥ 0.59. Item i8 (bodily pain) is a marker in both OHS and PHS. Item i11 (downhearted and blue) does not appear to be a good marker of overall, physical or mental health. However, its removal did not improve the model fit indices. We therefore decided to retain it to preserve the original length of the generic subscale. The ECV index of 0.57 indicates that, in our data, the common variance is more strongly accounted for by the general factor than by the specific factors. The ω_H_ coefficient of the general factor (0.71) indicates acceptable reliability and interpretability of the OHS score. In contrast, the ω_S_ coefficients for PHS and MHS are 0.33 and 0.21 respectively. Therefore, if OHS, PHS and MHS scores are reported, the interpretation of PHS and MHS as precise indicators of distinct constructs is limited. Beyond the statistical justification provided by the bifactor model, the use of the overall score can also be supported on conceptual and practical grounds. Conceptually, it reflects the holistic nature of HRQOL, which patients often perceive as a single construct. From a clinical and public health perspective, an overall score facilitates patient monitoring, communication of results, and international comparisons; however, domain-specific scores remain essential for capturing the multidimensionality of the construct.

The correlated three-factor model of the KDS, comprising BKD, SKD, and EKD, was supported by CFA, with sufficient goodness-of-fit indices. On the basis of modification indices and the conceptual overlap of items, the model included correlated error terms between the following item pairs: i20 (itchy skin) and i21 (dry skin); i29 (fluid restriction) and i30 (dietary restriction); i31 (your ability to work around the house) and i32 (your ability to travel); and i33 (being dependent on doctors and other medical staff) and i34 (stress or worries caused by kidney disease). This structural model has been extensively supported in the scientific literature [19,23]. Admittedly, the bifactor model shows better fit indices, but the general factor does not account for a substantial proportion of common variance (ECV = 22%), and its global score is neither reliable nor interpretable (ω_H_ = 0.13). This finding is in contrast to that reported by Peipert et al. in the US population [34]. These results, suggest that the KDS of the Senegalese KDQOL-36 should not be scored as a unidimensional measure. All items contribute significantly on their respective factors, with a loading greater than 0.40 (Figure 3), except for Item i28 (problems with your access/catheter site). These findings were consistent with those of Chen et al. in Chinese patients receiving maintenance dialysis [47] and Peipert et al. in the US patients [48].

All the scales and subscales were sufficiently internally consistent according to the COSMIN recommendations [26]. This funding is consistent with that found in the literature [19,23]. All the subscales demonstrated moderate to good test–retest reliability, with ICCs between 0.61 and 0.90, with only the PHS, BKD, SKD, and EKD subscales reaching the COSMIN-recommended threshold [26] for test–retest reliability. Bland–Altman analysis revealed that more than 5% of the observations fell outside the limits of agreement for all the subscales except the OHS, suggesting moderate test–retest agreement. These results indicate potential instability in responses over time or limited measurement reproducibility. However, the mean bias was low and not statistically significant for all the subscales.

The results of the known-groups analysis indicate that the Senegalese version of the KDQOL-36 discriminates well between patients based on sex and CKD stage. Specifically, patients on dialysis had significantly lower HRQOL scores, reflecting more impaired quality of life, compared with nondialyzed CKD patients, except for the mental health domain and burden. This finding is widely reported in the literature [7,9,11,12,49]. Similarly, women reported significantly lower HRQOL scores compared with men, again with the exception of the mental health domain, which is consistent with the findings of previous studies [9,11,12,50]. These findings support the good discriminant validity of the Senegalese version of the KDQOL-36.

In the 47 patients who rated their clinical condition as moderately to very much improved, HRQOL scores significantly increased between test and retest according to the Wilcoxon paired test, except for the PCS, MCS, and BKD subscales. In our opinion, the 15-day interval between test and retest may be too short to adequately assess responsiveness for dimensions such as burden and mental health. However, the SKD subscale appears to be particularly sensitive to change. This psychometric property has been evaluated in only a few validation studies of the KDQOL-36 [19,23]. The lack of floor/ceiling effects, except for the BKD subscale, further indicates that the instrument is capable of detecting changes across the full range of scores. The limited discriminant validity in the known-groups analysis of the kidney disease burden score, as well as its lack of responsiveness, may be partly explained by the observed floor effect.

Several clinical implications arise from this research. This study provides the Senegalese nephrology community with a validated instrument for measuring quality of life in CKD patients, particularly for use in clinical research. However, the use of the KDQOL-36 in routine clinical practice for quality-of-life assessment, particularly for patient follow-up, should be approached with caution and interpreted strictly in light of the patient’s clinical and biological context. The generic subscale of the instrument, corresponding to the SF-12, may also be used in other patient populations as well as in the Senegalese general population. In clinical research, we recommend the use of the overall health score and the three KDS scores. The PCS and MCS scores may also be used to allow comparisons with other populations. However, the PHS score and the MHS score, should be interpreted with caution.

This study has several strengths. Multiple psychometric properties were assessed using rigorous statistical analysis. This study included 575 patients, an adequate sample size according to the COSMIN recommendations [26]. The dataset is of high quality, with a low proportion of missing data. However, this study has several limitations that should be mentioned. The sample may not be representative of the broader Senegalese population living with CKD, as it included only patients who were followed in urban settings, and who were recruited with convenience sampling. Patients from rural areas may have more disadvantaged socioeconomic conditions. Furthermore, since not all Senegalese people are Wolof speakers, the instrument may be less appropriate for certain segments of the population. Although the bifactor model offers several advantages, it also has limitations, as discussed by Reise et al. [46]. The three-factor correlated model of the KDS showed lower CFI and TLI values than the predefined thresholds. However, the SRMR was below 0.08, and the widely accepted theoretical structure reported in the literature supported our choice. Responsiveness estimates may be sensitive to subjective global change ratings and the time horizon. Moreover, this property was not evaluated in the 10 clinically deteriorated patients because of the small sample size. The parameters used to assess responsiveness are considered inappropriate according to COSMIN [18] recommendations, although they have been documented in the scientific literature [51,52]. These measures are considered measures of the magnitude of change because of an intervention or other event rather than measures of the quality of the measurement instrument [18]. Responsiveness should be reassessed in a study including a larger sample, an objective assessment of clinical change (such as the occurrence of significant clinical events), and a longer interval between test and retest. Since measurement invariance was not demonstrated in this study, comparisons of quality-of-life levels between groups may be influenced by cultural, environmental, and personal factors differences, as well as by patients lived experiences. External validity was not assessed in this study because no quality-of-life questionnaire, whether generic or disease-specific, has yet been culturally adapted to the Senegalese context. Concurrent validity was not assessed because no gold standard for measuring quality of life in Senegalese CKD patients is available.

In conclusion, following content validation, the Senegalese version of the KDQOL-36 appears to be a valid and reliable tool for assessing HRQOL in patients with CKD. For the generic core, the OHS score is more interpretable. The instrument effectively discriminates between populations on the basis of their level of quality of life and demonstrated moderate to good reproducibility. Despite these limitations, the SKD and OHS, in particular, showed good responsiveness. Future work should include validation of the instrument using modern test theory, as well as an assessment of its measurement invariance across relevant subgroups.

## Supplementary Material

Supplementary file.docx

## Data Availability

The data that support the findings of this study are stored within the servers at the *Direction de l’Informatique et des Systèmes d’Information (DISI) de l’Université Cheikh Anta Diop* (UCAD) of Dakar, Senegal. These data are available upon reasonable request by contacting the corresponding author. The code used in the analyses is stored within servers at the *Centre d’Investigation Clinique – Epidémiologie Clinique* (CIC-EC, Université de Lorraine, Inserm, CHRU de Nancy, France) and can also be made available upon request. Any relevant summary statistics for the article are already included within the main article and the supplemental file and will be publicly available once the article is published.
