# Supplementary material for: Assessment of the psychometrics properties of the Senegalese kidney disease quality of life – 36 (KDQOL-36) in patients with chronic kidney disease
Source: Patient Relat Outcome Meas. 2026 Jun 22;17(1):49–61. doi: 10.1080/1179271X.2026.2688843 (PMC13536964; doi:10.1080/1179271X.2026.2688843)
Supplement: Supplementary file 1 [file DPRO_A_2688843_SM2557.docx]

**Supplementary data**

**Supplemental Text**

**Supplemental Text S1:** context and sociocultural adaptation

**Context**

This study concerned the content validation of the U.S. English version of the KDQOL-36 (1) within the Senegalese sociocultural context. According to COSMIN recommendations (2), when an existing instrument is used in a population different from the original target population for which it was developed, it is necessary to assess whether all items are relevant for the new population under study. Content validity can only be considered if the questionnaire is written in a language commonly spoken by the target population.

In Senegal, the main vehicular language is a dominant national language, Wolof. It is spoken by more than 70% (3) of the Senegalese population and serves as an interethnic communication language among speakers of different native languages (4). Calvet (4) also points out that Wolof is “the language of urban integration,” whereas French is “the language of integration into state institutions.” Thus, French, spoken by more than 26% of Senegalese people, together with Wolof, represents the two most predominant languages in Senegal (3). The intensive contact between these two languages has given rise to a unique linguistic context that must be considered when developing or adapting any measurement instrument. For this reason, the KDQOL-36 was translated into Wolof. The target population consists of patients living with chronic kidney disease (CKD).

**Sociocultural adaptation**

The cross-cultural adaptation process aimed to establish equivalence between the source version and the target version based on content validity (5). The original U.S. English version of the questionnaire was translated into Wolof by independent translators recruited through a call for applications. Three translators were selected from six candidates after CV review and telephone interviews. Two translators performed the forward translation from English to Wolof, and a third translator conducted the back-translation (Wolof to English), blinded to the original KDQOL-36 version.

The cross-cultural adaptation was supervised by an expert committee to assess semantic, idiomatic, experiential, and conceptual equivalence (7), ensuring adequate content validity. The committee comprised two nephrologists, one socio-anthropologist, one psychometrics expert, two epidemiologists, two translators, and two patient experts.

The process was conducted in several steps:

**Step 1: Translation**

Two translators independently produced two Wolof versions (VT1 and VT2).

**Step 2: First content validation meeting**

The two versions were compared during an expert committee meeting held on February 17, 2024, with the translators present. Consensus was reached to resolve discrepancies and produce a single reconciled version (VT12). In accordance with Epstein et al. (7), a back-translation of VT12 was then performed to facilitate communication with experts unfamiliar with the Wolof language.

**Step 3: Face validity**

Cognitive debriefing through individual interviews was conducted in March 2024 among 10 patients (5 men; mean age 44.75 years), recruited using non-random convenience sampling to assess item comprehension. This sample included eight chronic hemodialysis patients from the hemodialysis center of Aristide Le Dantec Hospital and two patients with CKD stages 3–4 followed on an outpatient basis at the nephrology department of Ouakam Military Hospital.

**Step 4: Second content validation meeting**

The expert committee met again on April 8, 2024, expanded to include additional nephrologists and the interviewer who conducted the cognitive debriefing, to validate the final version of the questionnaire.

This procedure led to the development of the Senegalese version of the KDQOL-36 in the Wolof language.

**References**

1. Kidney Disease Quality of Life Instrument (KDQOL) [en ligne]. [cité le 30 juill 2025]. Disponible sur: <https://www.rand.org/health-care/surveys_tools/kdqol.html>
2. Mokkink LB, Terwee CB, Knol DL, Stratford PW, Alonso J, Patrick DL, et al. The COSMIN checklist for evaluating the methodological quality of studies on measurement properties: A clarification of its content. BMC Med Res Methodol. 2010;10(1):22.
3. Hwang S. La place du français au Sénégal : portraits sociolinguistiques de la ville de Dakar. SHS Web Conf. 2024;191:02010.
4. Calvet LJ. Quel modèle sociolinguistique pour le Sénégal ? ou il n’y a pas que la véhiculante. Lang Société. 1994;68(1):89‑107.
5. Beaton DE, Bombardier C, Guillemin F, Ferraz MB. Guidelines for the Process of Cross-Cultural Adaptation of Self-Report Measures: Spine. 2000;25(24):3186‑91.
6. Herdman M, Fox-Rushby J, Badia X. A Model of Equivalence in the Cultural Adaptation of HRQoL Instruments: The Universalist Approach. Qual Life Res. 1998;7(4):323‑35.
7. Epstein J, Osborne RH, Elsworth GR, Beaton DE, Guillemin F. Cross-cultural adaptation of the Health Education Impact Questionnaire: experimental study showed expert committee, not back-translation, added value. J Clin Epidemiol. 2015;68(4):360‑9.

**Supplemental Text S2:** Scoring program of the overall health, physical health and mental health of the generic subscale.

The Overall Health (OHS), Physical Health (PHS), and Mental Health (MHS) subscale scores from the bifactor model were computed by weighting item responses by their standardized factor loadings on the corresponding specific factor (**Figure 1**). Only patients who answered at least 50% of the items within a subscale were included in the scoring. For example, for the OHS, if a patient answered fewer than 6 items, the score was considered missing and assigned a value of “NA”.

**Example of Scoring Procedure: Overall Health Subscale (OHS)**

1. **Raw score calculation:**

- Assign “**NA”** to the OHS score if **less than 50%** of the items are completed;
- Each item response is **multiplied by its standardized factor loading** on the OHS subscale = **weighted product;**
- The **sum of the weighted products** is computed for each individual and represents the **raw score;**

2. **Standardized score calculation, ranging from 0 to 100:**

- The extremes (minimum value, min and maximum value, max) are then calculated.
- The raw score is then transformed to a 0 – 100 scale using the following formula:

$$Standardized score = \frac{Raw score -min}{\max- min} \times100$$

**Supplemental Tables**

**Supplemental Table S1:** Definitions of the statistical indices used for interpreting the scale score of a confirmatory bifactor model. The definitions are taken from Rodriguez et al. (34).

| **Statistical indices** | **Definitions** | **Thresholds** |
| --- | --- | --- |
| Total omega (ω_T_) | a factor analytic model-based estimate of the reliability (true score variance over observed score variance) of unit-weighted test scores. Omega is the model-based analogue of coefficient alpha, except that it is appropriate for congeneric tests (varying factor loadings). | > 0.70 |
| Hierarchical Omega (ω_H_) | percent of total score variance attributable to a single general factor | > 0.50 |
| Subscale omega (ω_S_) | percent of subscale score variance attributable to a group factor, after removing the reliable variance due to the general factor | > 0.50 |
| Construct reliability (H) | quality of the measurement model defined by a particular set of items or how well the items reflect or account for the variance of the latent variable | > 0.80 |
| Factor determinacy (FD) | correlation between factor score estimates and factors | > 0.90 |
| Explained common variance (ECV) | percent of common variance explained by the general factor. This is a degree of unidimensionality index and is directly related to the relative strength of the general factor. | > 0.50 |
| Average Relative Parameter Bias (ARPB) | difference between an item’s loading in the unidimensional solution and its general factor loading in the bifactor (i.e., the truer model), divided by the general factor loading in the bifactor. | < 10% |

**Supplemental Table S2:** Baseline characteristics of the included and nonincluded patients**.**

| Characteristics | Overall, N = 662*** | Non-included, N = 87* | Included, N = 575*** | P**** |
| --- | --- | --- | --- | --- |
| Patient's age | 53.61 ± 15.37 | 49.48 ± 16.90 | 54.05 ± 15.15 | 0.034 |
| Missing | 25 | 25 | 0 |  |
| Dialysis duration | 53.00 (36.00, 90.00) | 48.00 (36.00, 120.00) | 53.00 (36.00, 84.00) | 0.5 |
| Missing | 185 | 40 | 145 |  |
| Dialysis center |  |  |  |  |
| Centre Alioune Badara Cisse | 132 (25) | 15 (20) | 117 (26) |  |
| Centre d’hémodialyse de Dakar | 47 (9.0) | 14 (19) | 33 (7.4) |  |
| CHN de PIKINE | 48 (9.2) | 1 (1.3) | 47 (11) |  |
| CHR de THIES | 63 (12) | 13 (17) | 50 (11) |  |
| CHU Aristide Le Dantec | 108 (21) | 19 (25) | 89 (20) |  |
| CHU Dalal Jamm | 23 (4.4) | 4 (5.3) | 19 (4.3) |  |
| Hôpital Militaire de Ouakam | 56 (11) | 3 (4.0) | 53 (12) |  |
| Hôpital Roi Baudoin | 45 (8.6) | 6 (8.0) | 39 (8.7) |  |
| Missing | 140 | 12 | 128 |  |
| Auto administration | 6 (0.9) | 0 (0) | 6 (1.0) | >0.9 |
| Missing | 8 | 6 | 2 |  |
| Men sex | 348 (53) | 39 (46) | 309 (54) | 0.2 |
| Missing | 2 | 2 | 0 |  |
| Education level (years) |  |  |  | 0.3 |
| 0 | 159 (25) | 11 (15) | 148 (26) |  |
| [1-6] | 132 (21) | 19 (27) | 113 (20) |  |
| [7-10] | 93 (15) | 10 (14) | 83 (15) |  |
| [11-13] | 121 (19) | 13 (18) | 108 (19) |  |
| > 13 | 134 (21) | 18 (25) | 116 (20) |  |
| Missing | 23 | 16 | 7 |  |
| Patient’s occupation |  |  |  | 0.005 |
| Formal occupation | 126 (21) | 15 (22) | 111 (21) |  |
| No formal occupation | 134 (23) | 5 (7.5) | 129 (25) |  |
| Without occupation | 327 (56) | 47 (70) | 280 (54) |  |
| Missing | 75 | 20 | 55 |  |
| Marital status |  |  |  | 0.13 |
| Single | 83 (13) | 11 (15) | 72 (13) |  |
| Divorced | 46 (7.3) | 7 (9.9) | 39 (7.0) |  |
| Married | 441 (70) | 42 (59) | 399 (72) |  |
| Widowed | 59 (9.4) | 11 (15) | 48 (8.6) |  |
| Missing | 33 | 16 | 17 |  |
| CKD stages |  |  |  | 0.048 |
| Stage 3-4 | 138 (21) | 11 (13) | 127 (22) |  |
| Stage 5 on dialysis | 523 (79) | 75 (87) | 448 (78) |  |
| Missing | 1 | 1 | 0 |  |
| Dialysis technic |  |  |  | >0.9 |
| Peritoneal dialysis | 12 (2.3) | 1 (1.3) | 11 (2.5) |  |
| Hemodialysis | 511 (98) | 74 (99) | 437 (98) |  |
| Missing | 139 | 12 | 127 |  |
| Diabetes mellitus | 121 (19) | 13 (19) | 108 (19) | 0.9 |
| Missing | 30 | 17 | 13 |  |
| Dialysis session number per week |  |  |  | 0.009 |
| 1 | 1 (0.2) | 0 (0) | 1 (0.2) |  |
| 2 | 49 (10) | 4 (7.0) | 45 (11) |  |
| 3 | 402 (83) | 43 (75) | 359 (84) |  |
| 4 | 32 (6.6) | 10 (18) | 22 (5.2) |  |
| Missing | 178 | 30 | 148 |  |
| Hemodialysis vascular access |  |  |  | 0.021 |
| Temporary catheter | 28 (5.8) | 3 (5.4) | 25 (5.9) |  |
| Permanent catheter | 123 (26) | 24 (43) | 99 (23) |  |
| Native arteriovenous fistula | 323 (67) | 29 (52) | 294 (69) |  |
| Graft arteriovenous fistula | 7 (1.5) | 0 (0) | 7 (1.6) |  |
| Missing | 11 | 11 | 0 |  |
| *^1^* Mean ± SD; n (%); median (IQR) | | | | |
| *^2^* Wilcoxon rank sum test; Pearson's Chi-squared test; Fisher's exact test  Abbreviations: CHN, centre hospitalier national; CHR, centre hospitalier régional; CHU, centre hospitalier universitaire ; | | | | |

**Supplemental Table S3**: Confirmatory factor analysis (CFA) results for the generic core and the kidney disease targeted subscale (KDS). Scaled TLI and CFI greater than 0.95 for all model except model 1, 2, and 3 of the generic subscale.

| **Model** | **_X_^2^/df** | **CFI** | **TLI** | **RMSEA** | **SRMR** |
| --- | --- | --- | --- | --- | --- |
| **Generic subscale** | | | | | |
| Model 1 | 25.0 | 0.632 | 0.541 | 0.210 (0.200-0.219) | 0.135 |
| Model 2 | 14.0 | 0.882 | 0.844 | 0.155 (0.145-0.165) | 0.100 |
| Model 3 | 24.7 | 0.640 | 0.534 | 0.209 (0.199-0.219) | 0.132 |
| Model 4 | 9.4 | 0.895 | 0.831 | 0.125 (0.113- 0.136) | 0.051 |
| Model 5 | 2.9 | 0.975 | 0.956 | 0.060 (0.048- 0.073) | 0.027 |
| **Kidney disease targeted subscale** | | | | | |
| Model 1 | 5.12 | 0.708 | 0.680 | 0.085 (0.080-0.090) | 0.09 |
| model 2 | 3.7 | 0.794 | 0.768 | 0.069 (0.064-0.074) | 0.078 |
| model 3 | 2.9 | 0.812 | 0.769 | 0.058 (0.053-0.063) | 0.059 |
| model 4 | 2.6 | 0.833 | 0.796 | 0.052 (0.047-0.057) | 0.056 |
| **Generic subscale** *model 1*, correlated two-factor model without correlated errors; *model 2*, correlated two-factor model with correlated errors terms between items 2 and 3, 4 and 5, and 9 and 10; *model 3*, correlated three-factor model; *model 4*, bifactor model without correlated errors; *model 5*, bifactor model with correlated errors terms between items 2 and 3, 4 and 5 and 11 and 12. **KDS**: *Model 1*, correlated three-factor model without correlated errors; *Model 2*, correlated three-factor model with correlated errors terms between items 20 and 21, 29 and 30, 31 and 32, and between 33 and 34; *model 3*, bifactor model without correlated errors; *Model 4*, bifactor model with correlated errors terms between items 31 and 32.  **Abbreviations**: df, degree of freedom; CFI, comparative fit index; TLI, Tucker Lewis Index; RMSEA, root mean square error of approximation; SRMR, standardized root mean square residual. | | | | | |

**Supplemental Table S4**: Statistical indices of the bifactor model of the generic subscale**.** The bifactor analysis of the Generic subscale assessed the structure and reliability of both total and specific scores using recommended indices. The general factor (Overall Health) explained 57% of the common variance (ECV = 0.57), with good overall reliability (*ω_T_* = 0.90) and well defined (H > 0.80 and FD > 0.90). The specific reliability of the total score was acceptable (ω_H_ = 0.71). The physical and mental scores were well-defined (H > 0.80 and FD > 0.90), but their unique variances (ω_S_ < 50*)* were insufficient to justify their use. The Overall Health score can be used reliably. However, the Physical and Mental Health scores should be interpreted with caution due to their low specific reliability.

| **Indices** | **OHS** | **PHS** | **MHS** |
| --- | --- | --- | --- |
| Total omega (ω_T_) | 0.90 | 0.86 | 0.77 |
| Hierarchical Omega (ω_H_) | 0.71 |  |  |
| Subscale omega (ω_S_) |  | 0.33 | 0.28 |
| Construct reliability (H) | 0.95 | 0.72 | 0.86 |
| Factor determinacy (FD) | 0.98 | 0.90 | 0.93 |
| Explained common variance (ECV) | 0.57 | 0.45 | 0.41 |
| Percent Uncontaminated Correlations (PUC) | 0.55 |  |  |
| Average Relative Parameter Bias (ARPB, %) | 0.94 |  |  |

**Supplemental Table S5**: Statistical indices of the bifactor model of the **kidney disease targeted subscale (KDS).** The bifactor analysis of the KDS assessed the structure and reliability of both total and specific scores using recommended indices. The general factor explained 22% of the common variance (ECV = 0.22), with good overall reliability (*ω_T_* = 0.83) but poorly defined (H < 0.80 and FD < 0.90). The specific reliability of the total score was very poor (ω_H_ = 0.13). The specific factors were well defined (H > 0.80 and FD > 0.90), and their unique variances were sufficient to justify their use (ω_S_ > 0.50). The burden, symptoms and effects scores should be interpreted reliably.

| **Indices** | **General factor** | **Burden** | **Symptoms** | **Effects** |
| --- | --- | --- | --- | --- |
| Total omega (*ω_T_)* | 0.83 | 0.81 | 0.79 | 0.78 |
| Hierarchical Omega (ω_H_) | 0.13 |  |  |  |
| Subscale omega (*ω_S_)* |  | 0.67 | 0.76 | 0.69 |
| Construct reliability (H) | 0.74 | 0.83 | 0.85 | 0.83 |
| Factor determinacy (FD) | 0.89 | 0.92 | 0.94 | 0.94 |
| Explained common variance (ECV) | 0.22 | 0.80 | 0.84 | 0.71 |
| Percent Uncontaminated Correlations (PUC) | 0.64 |  |  |  |
| Average Relative Parameter Bias (ARPB, %) | 7.05 |  |  |  |

**Supplemental Figures**

**Supplemental Figure S1**: Study flow chart.

**Supplemental Figure S2:** Bland–Altman plot. Assessment of the agreement between test (t0) and retest (t1) scores in 74 patients who rated their clinical condition as stable. OHS, Overall Health Scale; PHS, Physical Health Scale; MHS, Mental Health Scale; PCS, Physical Component Summary; MCS, Mental Component Summary. The subscale scores were standardized between 0 and 100, with the higher scores indicating better quality of life.

**Supplemental Figure S3**: Box plot of KDQOL-36 subscale scores at test (T0) and retest (T1) in 47 patients whose clinical condition improved moderately to very much. The subscales scores were expressed as standardized score ranging from 0 to 100 with higher scores indicating better quality of life. OHS, Overall health Scale; PHS, Physical Health Scale; MHS, Mental Health Scale; PCS, Physical Component Summary; MCS, Mental Component Summary
